# Bewildering ST-Elevation With Wellens’ Electrocardiogram Pattern – Is Myocarditis in Your Differentials?

**DOI:** 10.7759/cureus.12983

**Published:** 2021-01-29

**Authors:** Ali Hussain, Mubashar Iqbal, Gondal Mohsin, Hassan A Mirza, Muhammad Talha Butt

**Affiliations:** 1 Acute Medicine, Pinderfields General Hospital, Wakefield, GBR; 2 Respiratory Medicine, Sheffield Teaching Hospitals NHS Foundation Trust, Sheffield, GBR; 3 Cardiology, Sheffield Teaching Hospitals NHS Foundation Trust, Sheffield, GBR; 4 Acute Medicine, Sheffield Teaching Hospitals NHS Foundation Trust, Sheffield, GBR; 5 Acute Medicine, Pinferfields General Hospital, Wakefield, GBR

**Keywords:** myocardial infarction, echocardiography, electrocardiogram, myocarditis, cardiac magnetic resonance imaging, late gadolinium enhancement, st-elevation myocardial infarction (stemi), primary percutaneous coronary intervention

## Abstract

Myocarditis is the inflammation of the myocardium and is a challenging diagnosis owing to the heterogeneity in its etiology, pathogenesis and clinical presentations. It often presents as an acute coronary syndrome (ACS) mimic and hence may pose both diagnostic and therapeutic challenges to treating physicians to reliably differentiate between these two entities. In this case, we discuss a young male whose initial presentation of chest pain was dubious of the acute coronary syndrome but detailed history, physical examination and by careful selection of non-invasive investigations including echo and cardiac magnetic resonance imaging (MRI), led to a diagnosis of acute myocarditis. This approach not only avoided undue radiation exposure to a young individual but also eluded the unnecessary treatment with potent antiplatelet and anticoagulation therapy.

## Introduction

Myocarditis is an inflammatory condition of heart muscle whose clinical manifestation ranges from minor non-specific symptoms (like fever, myalgias, lethargy) to fulminant presentations (like acute pulmonary edema, cardiogenic shock) which can prove to be catastrophic. Acute chest pain remains the most prevalent symptom with myocarditis and can prove to be an arduous task to exclude it from the ischemic pain associated with the acute coronary syndrome (ACS) [1]. Wellen’s type ECG pattern has been classically described in relation to an ischemic lesion involving left descending artery territory. The most narrated ECG findings with acute myocarditis include diffuse ST elevation or ST depression, but Wellen’s type ECG has not been much associated with myocarditis in the literature [1]. Our case highlights the importance of widening the physician’s differential diagnostic list to include acute myocarditis in patients exhibiting pseudo-Wellen’s pattern on ECG. This can prevent patients to get unduly irradiated in the cardiac catheterization lab via the primary percutaneous coronary intervention (PPCI) pathway when the diagnosis could have been confirmed non-invasively via cardiac MRI. It can also formidably avoid complications associated with the use of strong antiplatelet and anticoagulant medications routinely used otherwise in ACS patients.

## Case presentation

A 19-years-old male presented to the emergency department (ED) with three days’ history of constant central chest pain, which was sudden in onset, crushing in character, 9/10 in intensity, and non-radiating. The pain was worsened by taking a deep breath and lying down but slightly improved by leaning forward. There was no associated nausea, vomiting, sweating, palpitations, dizziness, or syncope. Physical exertion had no impact on pain. He denied any history of fever, coryzal symptoms, skin rash, cough, hemoptysis, or breathlessness. There was no history of recent travel or chest trauma. There was no history of anxiety and the rest of his past medical history was also insignificant. He was a student and denied any recent consumption of tobacco or illicit drugs like cocaine or amphetamine. There was no family history of ischemic heart disease.

On arrival to ED, his vitals were stable (respiratory rate 18, a saturation of O_2_ was 99% on room air, blood pressure (BP) 131/71, heart rate 86 beats per minute (bpm), and temperature 37 °C). He was alert and oriented but appeared to be in agony. The patient was of average body mass index (BMI) and did not have any marfanoid features. On cardiovascular examination, heart sounds were normal and there was no pericardial rub audible. There was no difference in BP recordings in both arms. Auscultation of the chest was normal and there was no evidence of calf swelling or tenderness. The rest of the systemic examination was normal.

**Figure 1:**
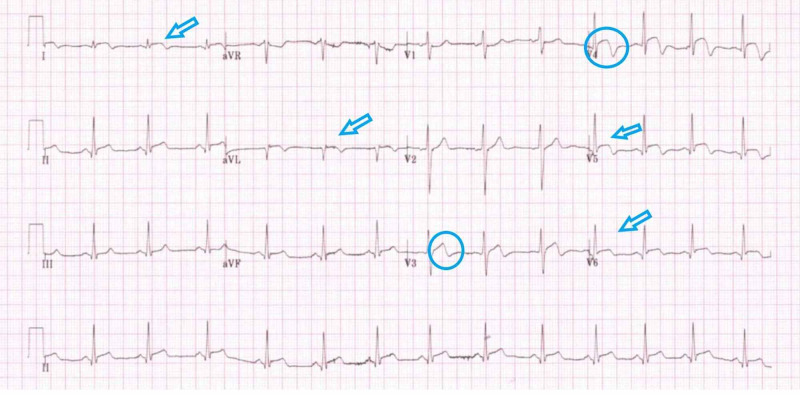
Diffuse concave upwards ST-elevation in both chest and limb leads (arrows) with biphasic T-waves (Wellen’s pattern) in anterior leads (circles) which raised concerns about STEMI. STEMI: ST-elevation myocardial infarction.

His 12 leads electrocardiogram (Figure 1) on presentation, showed diffuse concave upwards ST-elevation in both chest and limb leads with biphasic T-waves (Wellen’s pattern) in anterior leads which raised concerns about ST-elevation myocardial infarction (STEMI). Given his ongoing chest pain, he was initially referred to the PPCI center but was advised to treat him under local cardiologist care. An immediate transthoracic echo was performed which showed a normal biventricular systolic function without any regional wall motion abnormalities (RWMA). There were no valvular or structural abnormalities detected. Chest X-ray did not reveal any signs of effusion, pneumothorax, pneumomediastinum, or any widened mediastinum. His biochemistry labs showed markedly elevated troponin levels as 2038 ng/L (NR 0-15 ng/L). The rest of the blood tests are tabulated in Table 1 and were also unremarkable.

**Table 1:** Illustrates the baseline labs for the patient Hb: haemoglobin, WCC: white blood cells, eGFR: estimated glomerular filtration rate, PCR: polymerase chain reaction.

Investigation	Normal Value	Result
Hb g/L	130–180	153
WCC 10^9^/L	4–13	9.0
Platelets 10^9^/L	135–400	227
Lymphocytes 10^9^/L	1.0–4.0	1.80
Sodium (mmol/L)	135	141
Potassium (mmol/L)	3.0	4.6
Urea (µmol/L)	2.5	4.1
Creatinine	61	80
eGFR mL/min/1.73 m^2^		123
Calcium mmmol/L	2.20–2.60	2.46
Troponin I ng/L	0–15	2038
Mg	0.7–1.00	0.91
Covid PCR	Negative	Negative

**Figure 2:**
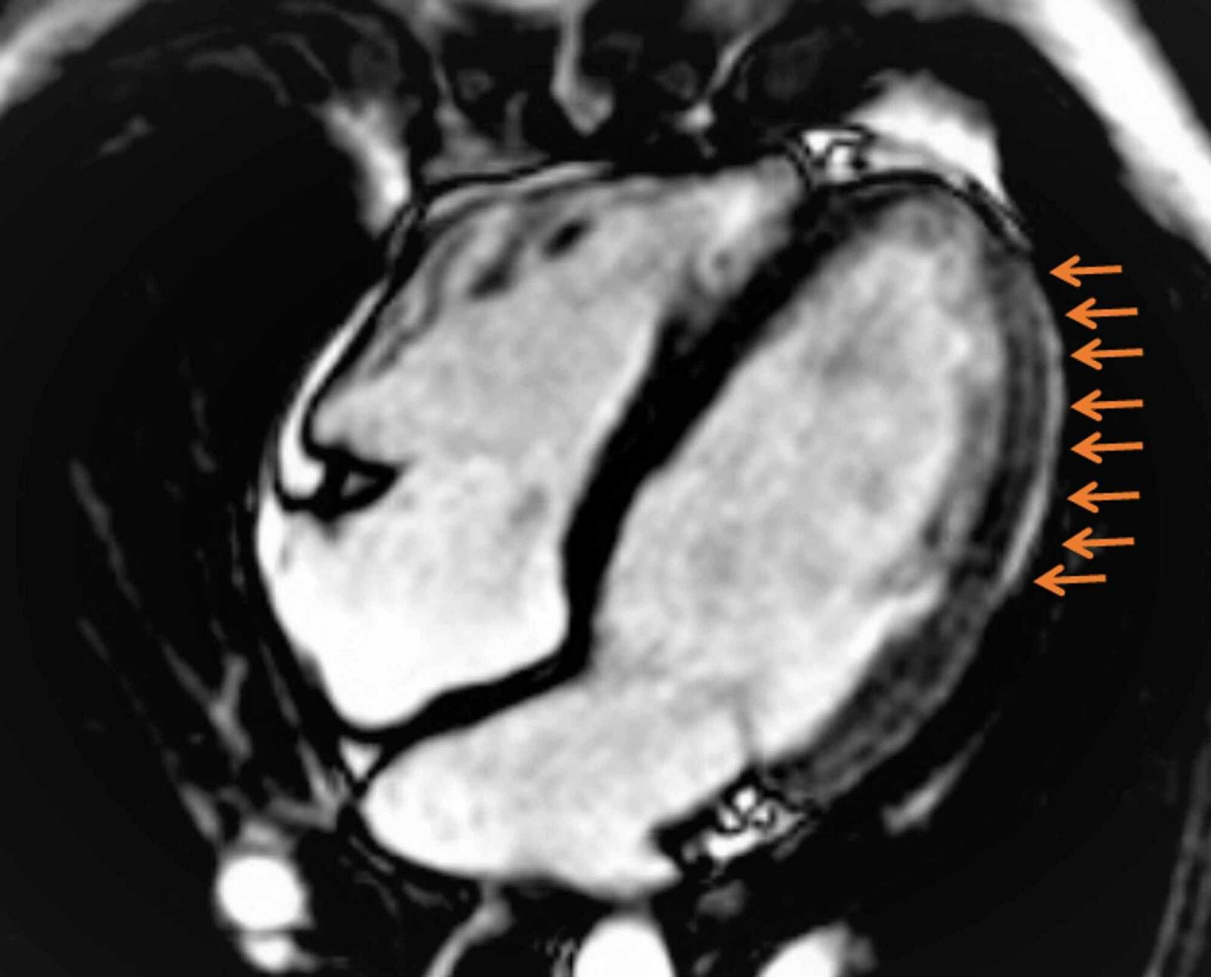
Cardiac MRI showing extensive sub-epicardial lateral wall LGE (shown by arrows) in keeping with myocarditis. MRI: magnetic resonance imaging, LGE: late gadolinium enhancement.

Based on his overall clinical presentation, ECG findings, lack of risk factors for ischemic heart disease, and absence of RWMA on echo, he was admitted to the coronary care unit (CCU) for close monitoring. It was decided to proceed directly to cardiac magnetic resonance imaging (MRI) rather than performing an invasive angiogram as he fulfilled the clinical criteria for myocarditis. This strategy proved to be rewarding in terms of making a diagnosis of acute myocarditis and cardiac MRI (Figure 2) that confirmed the presence of extensive subepicardial late gadolinium enhancement (LGE) in the basal to mid-lateral, mid inferior, and apical segments which correlated well with ST-segment changes on the ECG.

## Discussion

Chest pain is one of the most common complaints with which patients present to the ED. Approximately 8 million people visit ED annually in the United States [2]. In younger patients less than 40 years of age, the prevalence of ACS is estimated to be less than 1%; however, it cannot be completely ignored [3]. Therefore, chest pain in younger patients could be multi-aetiological and may present with a multitude of signs and symptoms. Anatomically, any diseased organ in the chest can lead to chest pain ranging from heart to large vessels, aorta and pulmonary trunk, lungs, esophagus, stomach, mediastinum, and abdominal viscera. Hence, the main focus should always remain to exclude the life-threatening causes of chest pain which are mentioned in Table 2.

**Table 2:** Life-threatening causes of chest pain.

Anatomical Structure	Causes
Cardiac	Acute coronary syndrome, pericardial tamponade, and myocarditis
Aorta	Aortic dissection
Pulmonary	Pulmonary embolism
Pleura	Tension pneumothorax
Oesophagus	Boerhaave syndrome
Mediastinum	Mediastinitis

Acute myocarditis is amongst the infrequent causes of chest pain which can masquerade as an acute coronary syndrome. Although viral infection remains the most common cause of myocarditis, however in a few cases, there is no history of preceding viral prodrome as in our described case. This makes it even harder to be certain regarding the penultimate diagnosis, and hence, requires experienced eyes to decide on the further route of investigations. An accurate diagnosis is vital, as prognosis and treatment may vary according to the cause and hemodynamic status of the patient. Furthermore, a proportion of these patients get referred to PPCI centers as STEMI for consideration of PCI or may even receive thrombolysis if the PPCI center is more than two hours away. As a result, they may get inappropriately exposed to complications related to invasive coronary angiography, radiations, and thrombolysis, all of which could be avoidable with a non-invasive approach.

Our case fulfilled both clinical and diagnostic criteria (Table 3) for myocarditis as per ESC guidance which included ischemic-mimic chest pain, ECG changes (ST-changes), raised troponin, and cardiac MRI evidence of sub-epicardial enhancement.

**Table 3:** ESC criteria for acute myocarditis. Diagnostic criteria for clinically suspected myocarditis. Adapted from a position statement of the European Society of Cardiology Working Group on Myocardial and Pericardial Diseases (European Heart Journal (2013)). MRI: magnetic resonance imaging, ECG: electrocardiogram, ESC: European Society of Cardiology, RV: right ventricle, LV: left ventricle, Tnl: troponin I, TnT: troponin T, LGE: late gadolinium enhancement.

Clinical Presentations
Acute chest pain, pericarditis, or pseudo-ischemic new-onset (days up to three months) or worsening of: dyspnoea at rest or exercise, and/or fatigue, with or without left and/or right heart failure signs subacute/chronic (three months) or worsening of: dyspnoea at rest or exercise, and/or fatigue, with or without left and/or right heart failure signs, palpitation, and/or unexplained arrhythmia symptoms and/or syncope, and/or aborted sudden cardiac death, unexplained cardiogenic shock
Diagnostic Criteria
(i) ECG/Holter/stress test features
Newly abnormal 12 lead ECG and/or Holter and/or stress testing, any of the following: I- to III-degree atrioventricular block, or bundle branch block, ST/T wave change (ST elevation or non-ST elevation, T-wave inversion), sinus arrest, ventricular tachycardia or fibrillation and asystole, atrial fibrillation, reduced R-wave height, intraventricular conduction delay (widened QRS complex), abnormal Q-waves, low voltage, frequent premature beats, supraventricular tachycardia.
(II) Myocardiocytolysis markers elevated TnT/TnI
(III) Functional and structural abnormalities on cardiac imaging (echo/angio/cardiac MRI)
New, otherwise unexplained LV and/or RV structure and function abnormality (including incidental finding in apparently asymptomatic subjects): regional wall motion or global systolic or diastolic function abnormality, with or without ventricular dilatation, with or without increased wall thickness, with or without pericardial effusion, with or without endocavitary thrombi.
(IV) Tissue characterization by cardiac MRI
Oedema and/or LGE of classical myocarditic pattern.

**Figure 3:**
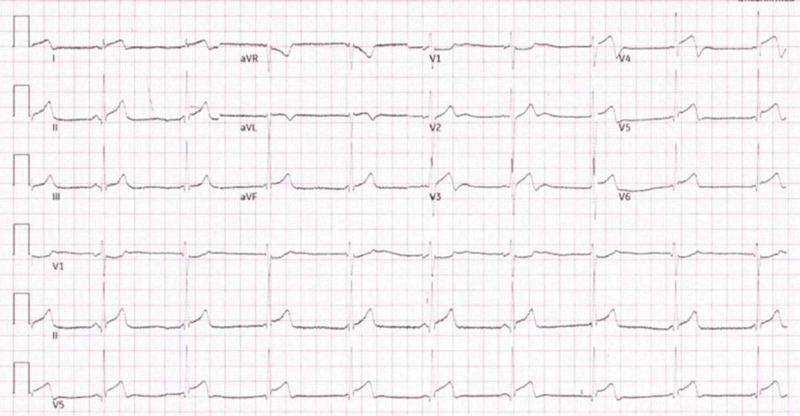
Absence of dynamic changes and pathological Q-waves favors myocarditis (compare with ECG 1).

Acute myocarditis and STEMI may sometimes be clinically indistinguishable, and hence, it becomes remarkably difficult to make a concrete diagnosis for both emergency physicians and cardiologists alike. However, there are subtle features that can help to delineate both conditions. History of young age without risk factors for coronary artery disease (CAD), preceding flu-like symptoms, all favors non-coronary cause [4]. Moreover, ST-elevation in myocarditis is usually diffuse and usually does not correspond to a particular vascular territory. In contrast, ST-changes follow a vascular territorial distribution pattern. To complicate the matter, Wellen’s type ECG pattern (commonly associated with ischemia), may be present in myocarditis as in the described case, albeit less described in the medical literature. Serial ECGs, to visualize any dynamic changes, and development of Q-waves could point towards STEMI which were absent in our case (Figure 3).

An echocardiogram can be used as a surrogate marker to detect any new RWMA as a readily available modality of imaging. Cardiovascular MRI is a non-invasive imaging tool that can reliably confirm the diagnosis of myocarditis. Subepicardial late gadolinium enhancement is the pathognomonic feature of acute myocarditis [5]. Lake Louise criteria for cardiac MRI is widely used for the diagnosis of myocarditis in suspected patients [6-8]. Friedrich et al. [6] showed its accuracy in a subgroup of patients presented with "infarct-like" presentation including elevated troponin level, chest pain, and ST-segment elevation, as in our patient.

European Society of Cardiology (ESC) also recommends consideration of invasive endomyocardial biopsy (EMB) in patients with clinically suspected myocarditis [7,8]; however, this technique has limited sensitivity in the presence of patchy involvement. Due to risks associated with EMB, it is only reserved in cases where there are concerns about clinical improvement, expected ongoing inflammatory process, or when the diagnosis is uncertain [8].

## Conclusions

Myocarditis is an important differential diagnosis of acute myocardial infarction. The presence of Wellen’s type ECG should also prompt one to think of the possibility of myocarditis in a patient with a low-risk factor profile for ischemic insult. Its noteworthy diffuse ST-elevation without vascular territory distribution favors myocarditis over acute coronary syndrome. Moreover, cardiac MRI may be utilized as a non-invasive tool to differentiate myocarditis from myocardial infarction.

## References

[1] Cooper LT (2009). Myocarditis. N Engl J Med.

[2] Owens PL, Barrett ML, Gibson TB, Andrews RM, Weinick RM, Mutter RL (2010). Emergency department care in the United States: a profile of national data sources. Ann Emerg Med.

[3] Gudiño Gomezjurado A, Pujol Freitas B, Contreira Longatto F, Negrisoli J, Aguiar Sousa G (2017). Acute coronary disease, prognosis and prevalence of risk factors in young adults. Medwave.

[4] Nozari Y, Tajdini M, Mehrani M, Ghaderpanah R (2016). Focal myopericarditis as a rare but important differential diagnosis of myocardial infarction: a case series. Emergency (Tehran).

[5] Testani JM, Kolansky DM, Litt H, Gerstenfeld EP (2006). Focal myocarditis mimicking acute ST-elevation myocardial infarction: diagnosis using cardiac magnetic resonance imaging. Tex Heart Inst J.

[6] Friedrich MG, Sechtem U, Schulz-Menger J (2009). Cardiovascular magnetic resonance in myocarditis: a JACC white paper. J Am Coll Cardiol.

[7] Laissy JP, Messin B, Varenne O, Iung B, Karila-Cohen D, Schouman-Claeys E, Steg PG (2002). MRI of acute myocarditis: a comprehensive approach based on various imaging sequences. Chest.

[8] Alida C, Sabine P, Eloisa A (2013). Current state of knowledge on aetiology, diagnosis, management, and therapy of myocarditis: a position statement of the European Society of Cardiology Working Group on Myocardial and Pericardial Diseases. Eur Heart J.

